# Associations of clinical subtypes and bile acid levels of intrahepatic cholestasis of pregnancy with pregnancy outcomes

**DOI:** 10.1038/s41598-024-63183-9

**Published:** 2024-05-28

**Authors:** Fan Feng, Juhong Li, Junqun Liao, Shiyi Qin, Yaling Liu, Xian Che, Yanjun Zhou, Dan Jiang, Huiqin Xiao, Aixing Chen, Yong Shao

**Affiliations:** 1 Department of Obstetrics and Gynecology, The First Affiliated Hospital of Chongqing Medical University, Chongqing, 400016 China; 2 Department of Experiment, The First Affiliated Hospital of Chongqing Medical University, Chongqing, China; 3 Department of Obstetrics and Gynecology, Yubei District Maternal and Child Health Hospital, Chongqing, China

**Keywords:** Intrahepatic cholestasis of pregnancy, Pregnancy outcome, Subtype of ICP, Risk factors, Predictive markers

## Abstract

Intrahepatic cholestasis of pregnancy (ICP) can lead to many adverse pregnancy outcomes, and the influencing factors remain unclear at present. This study retrospectively analyzed clinical data from 1815 pregnant women with ICP and evaluated the relationship between ICP subtypes, gestational age at onset, and pregnancy outcomes. The results of this study show that during pregnancy, the levels of biochemical indicators (TBA, DBIL and ALT) in the serum of pregnant women initially diagnosed with subtypes of ICP were noted to constantly change, and the subtype of ICP and its severity also changed. The incidence of adverse pregnancy outcomes [meconium-stained amniotic fluid (MSAF), NICU transfer, Apgar score ≤ 7 at 1 min, and preterm birth] in patients with ICP1 (icteric type) was significantly higher than for patients with ICP2, ICP3 or ICP4. The preterm birth rate of early-onset ICP was higher than that of late-onset ICP in ICP1 and ICP3 subtypes. In conclusion, the outcome of pregnancy in women with ICP is closely related to the serum TBA level and ICP subtype, which should be recognized in the clinic.

## Introduction

Intrahepatic cholestasis of pregnancy (ICP) is a pregnancy-specific complication often occurring in the second and third trimesters of pregnancy. It is clinically characterized by skin pruritus and elevated fasting serum total bile acid (TBA) (≥ 10 μmol/L), possibly accompanied by elevated liver enzymes, which can quickly disappear or return to normal after delivery. ICP can increase the morbidity and mortality of perinatal diseases, mainly including premature birth, fetal distress and stillbirth^1^. The morbidity of ICP is associated with race and region. It is more prevalent in Scandinavia and South America, but only 0.1–1.5% in Europe, the United States, Canada and Australia^2^. In China, the rate of ICP is 1.2%^3^, but it is as high as 4–10% in Chongqing, Sichuan and Yangtze River Basin regions^4–6^.

The pathogenesis of ICP remains unclear, possibly influenced by genetic and environmental factors. The clinical diagnosis of ICP is dependent on the maternal serum TBA level, and correlations between a higher TBA level and fetal complications have been reported. In 2019, Ovadia et al.^7^ found a significantly increased risk of stillbirth with TBA ≥ 100 μmol/L. In 2021, ICP was classified by the Society for Maternal–Fetal Medicine (SMFM) into three risk levels based on the maternal serum TBA level: low risk (10 ≤ TBA < 40 μmol/L), moderate risk (40 ≤ TBA < 100 μmol/L) and high risk (TBA ≥ 100 μmol/L)^8^. In the 2022 Royal College of Obstetricians and Gynecologists (RCOG) Green-top Guidelines, ICP was classified into mild type (19 ≤ TBA < 40 μmol/L), moderate type (40 ≤ TBA < 100 μmol/L) and severe type (TBA ≥ 100 μmol/L) based on maternal serum level^9^.

At present, ursodeoxycholic acid (UDCA) is the first-line drug for the clinical treatment of ICP. Glantz et al. found that UDCA can reduce some biochemical markers in ICP pregnant women with serum bile acids ≥ 40 μmol/L, but not on fetal complication rates^10^. Ovadia et al. found that UDCA treatment have no significant effect on the prevalence of stillbirth in women with ICP. The composite outcome of stillbirth and preterm birth was reduced when the analysis was restricted to RCTs, whereas the risk of preterm birth was reduced when only singleton births were considered, especially among women with BA ≥ 40 μmol/L. Therefore, it is of great importance to further investigate the factors influencing fetal outcomes in ICP.

As previously reported, pregnant women with ICP have unique metabolic patterns regarding their serum TBA profile. In our previous work, patients with ICP were divided into four subtypes based on serum TBA, direct bilirubin (DBIL), and alanine aminotransferase (ALT) levels. The incidence of meconium-stained amniotic fluid (MSAF), premature birth and cesarean section in patients with ICP1 was significantly higher than for patients with either ICP2, ICP3 or ICP4, suggesting that the levels of serum TBA, DBIL and ALT in pregnant women are the key indicators affecting pregnancy outcome^11^.

In the present study, the clinical characteristics and outcomes for patients with different subtypes of ICP were analyzed in a larger cohort than in previous studies, and correlations between early-onset ICP (time of initial onset < 28 gestational weeks^12^) and pregnancy outcomes was also analyzed.

## Results

### Dynamic changes in ICP subtypes during the follow-up period of pregnant women with ICP

**Figure 1:**
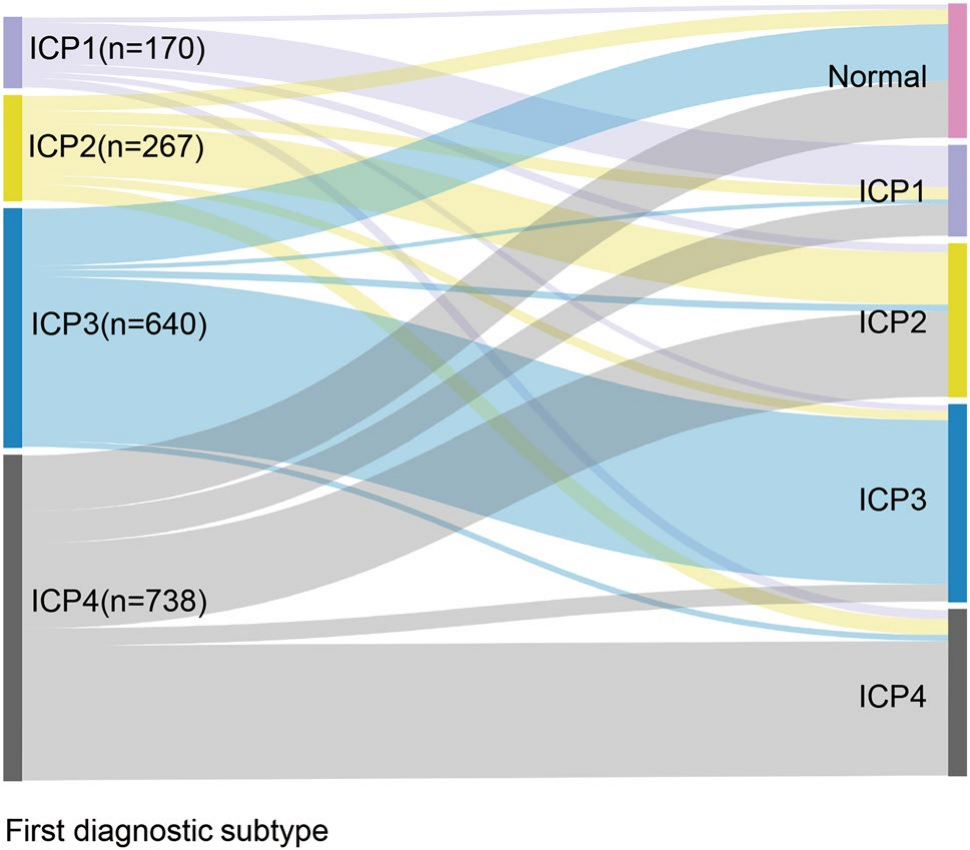
Dynamic changes in ICP subtypes during the follow-up period of pregnant women with ICP.

Based on the biochemical characteristics of serum TBA, DBIL and ALT at the time of initial diagnosis, pregnant women with ICP were divided into four groups, ICP1, ICP2, ICP3 and ICP4, as described in the “Methods”. During the follow-up period (from the initial diagnosis to delivery), the patients were further divided into normal, ICP1, ICP2, ICP3 and ICP4 groups according to the changes in serum biochemical indicators (Table S1 and Fig. 1). The results revealed that during the follow-up period, the serum biochemical indicators returned to normal in only 8.8% of cases, whereas 74.1% of cases remained in ICP1, but 14.7%, 10.6% and 16.5% changed from ICP1 to ICP2, ICP3 and ICP4, respectively (n = 170). In the ICP2 group (n = 267), serum biochemical indicators returned to normal in 17.2% of cases, whereas 59.6% of remained in ICP2, and 13.9%, 10.1% and 18.0% changed to ICP1, ICP3 and ICP4, respectively. In the ICP3 group (n = 640), these indicators returned to normal in 27.0% of cases, with 77.7% remaining in ICP3, and 2.0%, 3.3% and 2.8% changing to ICP1, ICP2 and ICP4, respectively. Finally, in the ICP4 group (n = 738), serum biochemical indicators returned to normal in 23.0% of patients, 55.4% remained in ICP4, and 13.1%, 35.1% and 7.2% changed to ICP1, ICP2 and ICP3, respectively. These results document that during pregnancy, the levels of serum biochemical indicators (TBA, DBIL and ALT) in pregnant women initially diagnosed with subtypes of ICP were constantly changing, and the subtype of ICP and its severity also changed. In particular, although the serum TBA level in pregnant women with ICP4 was normal at the time of initial diagnosis, in some patients it could still rise during pregnancy, which requires attention.

### Pregnancy outcomes for women with subtypes of ICP

**Figure 2:**
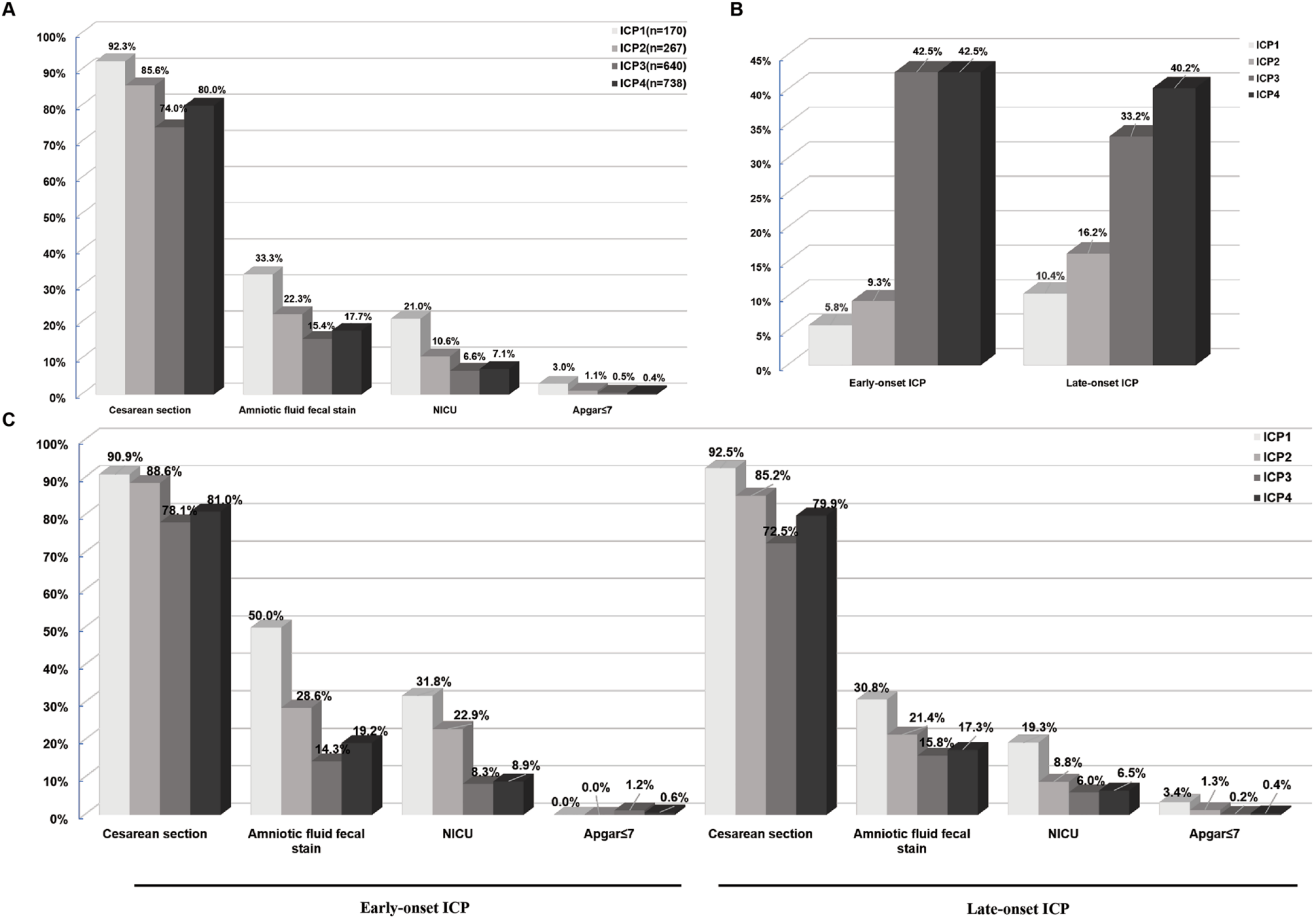
Pregnancy outcomes for women with subtypes of ICP. (**A**) Pregnancy outcomes of different subtypes in total ICP cases; (**B**) Proportion of different subtypes in early-onset and late-onset ICP; (**C**) Pregnancy outcomes for women with different subtypes of early- or late-onset ICP.

The pregnancy outcomes for women with different subtypes of ICP were analyzed (Table S2, Fig. 2A). The results showed that ICP subtype 1 had an increased risk of preterm birth, especially iatrogenic preterm birth, compared with other subtypes. The preterm birth rate of ICP2 was also significantly higher than that of ICP3 and ICP4. The proportion of MSAF cases in the ICP1 group (33.3%) was significantly higher than in ICP2 (22.3%), ICP3 (15.4%) or ICP4 (17.7%). The proportion of neonates transferred to the NICU in the ICP1 group (21.0%) was also significantly higher than in ICP2 (10.6%), ICP3 (6.6%) or ICP4 (7.1%). Finally, the proportion of neonates with an Apgar score ≤ 7 at 1 min in the ICP1 group (3.0%) was significantly higher than in the ICP2, 3 or 4 groups (1.1%, 0.5% 0.4%, respectively). There was no significant difference in 5 min Apgar score < 7 and 1 min Apgar score < 4 between subgroups, but our analysis was probably limited by the low overall event rate. These results show that the incidence of adverse pregnancy outcomes (defined as meconium-stained amniotic fluid, NICU transfer, and Apgar score ≤ 7 at 1 min, preterm birth) in patients with ICP1 (icteric type) was significantly higher than for ICP2, ICP3 and ICP4. Thus, adverse pregnancy outcomes for women with ICP are closely related to the subtype of ICP (icteric type or not).

### Pregnancy outcomes for women with different subtypes of early- or late-onset ICP

According to the time of initial onset, patients with ICP were divided into early-onset or late-onset groups. In the former (398/1815, 21.9%, time of initial onset < 28 gestational weeks), there were 5.7%, 9.3%, 42.5% and 42.5% of ICP1, ICP2, ICP3 and ICP4 cases, respectively. In the latter (1417/1815, 78.1%, time of initial onset ≥ 28 gestational weeks), these proportions were 10.4%, 16.2%, 33.2% and 40.2%, respectively (Table S3, Fig. 2B).

Among pregnant women with early-onset ICP, of the four groups, the gestational age at delivery was the earliest and the neonatal weight was the lowest in the ICP1 group. The preterm birth rate of ICP1, especially iatrogenic preterm birth, was higher than that of ICP3 and ICP4. The incidence of MSAF in patients with ICP1 (50%) was significantly higher than for ICP2 (28.6%), ICP3 (14.3%) or ICP4 (19.2%). The NICU transfer rate in the ICP1 group (31.8%) was also significantly higher than for ICP2 (22.9%), ICP3 (8.3%) and ICP4 (8.9%), but there were no significant differences in the Apgar score at 1 min among the four groups.

For pregnant women with late-onset ICP, the gestational age at delivery was again the earliest and the neonatal weight was the lowest for patients in the ICP1 group. ICP1 had the highest rates of preterm birth and iatrogenic preterm birth compared with the other subtypes. The preterm birth rate of ICP2, especially iatrogenic preterm birth, was higher than that of ICP3. The incidence of MSAF in the ICP1 group (30.8%) was significantly higher than in the ICP2 (21.4%), ICP3 (15.8%) and ICP4 (17.3%) groups. The NICU transfer rate in the ICP1 group (19.3%) was also significantly higher than in ICP2 (8.8%), ICP3 (6.0%) and ICP4 (6.5%) groups. In this instance, the proportion of neonates with an Apgar score ≤ 7 at 1 min in the ICP1 group (3.4%) was also significantly higher than in the ICP2 (1.3%), ICP3 (0.2%) or ICP4 (0.4%) groups (Table S3, Fig. 2C). It can be seen that the pregnancy outcome was poorer in ICP1 (icteric type) group for both early-onset ICP and late-onset ICP, indicating that the outcome is closely related to the subtype of ICP (icteric type or not).

### Association between gestational week and pregnancy outcome for pregnant women with early- or late-onset ICP

To determine the influence of gestational week at onset on pregnancy outcomes for patients with ICP, patients in each ICP group were divided into those with early- or late-onset ICP in order to exclude any interference of subtype with pregnancy outcome. In ICP1, preterm birth rates were higher in early-onset ICP than in late-onset ICP. In ICP3, the preterm birth rate of early-onset ICP, especially iatrogenic preterm birth, was higher than that of late-onset ICP. Among pregnant women with ICP1 or ICP3, the gestational age at delivery was earlier and the neonatal weight was lower in those with early-onset relative to late-onset ICP. Among patients with ICP2, the proportion of neonates transferred to the NICU was higher in early-onset than in late-onset ICP. However, there were no significant differences between early-onset and late-onset ICP groups among pregnant women with ICP4 (Table S4).

### Association between serum TBA level and pregnancy outcome

**Figure 3:**
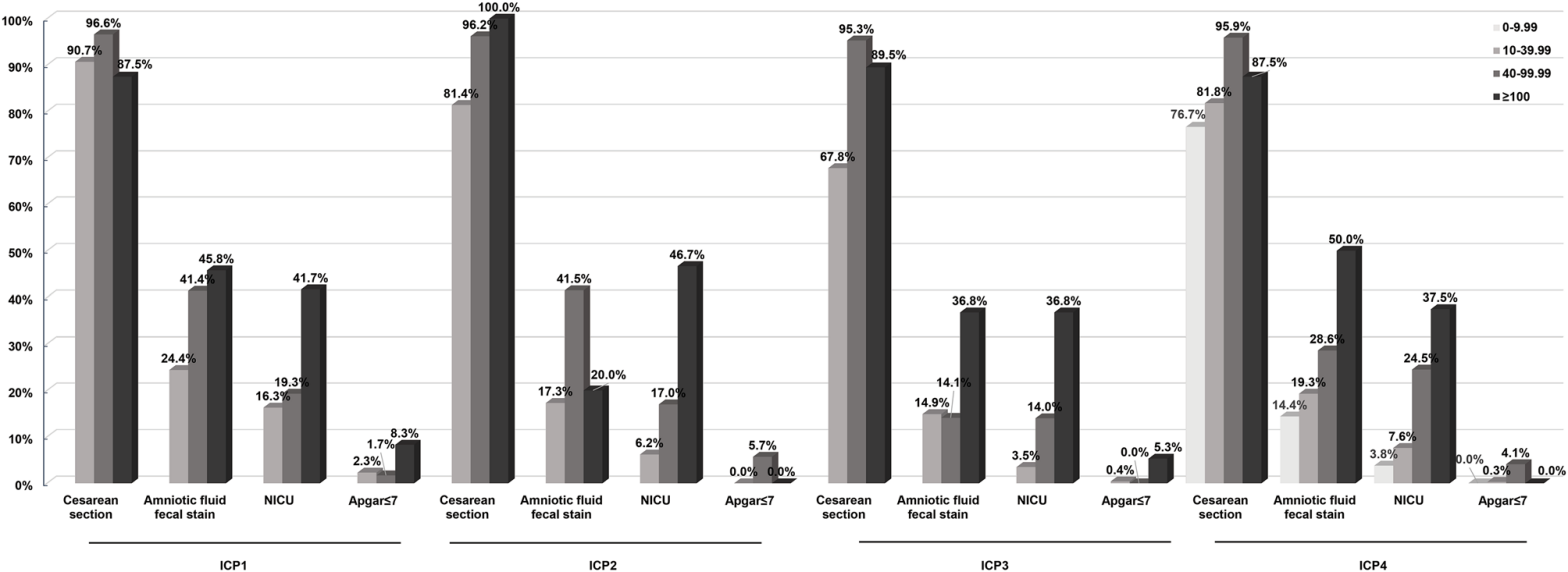
Association between serum TBA level and pregnancy outcome.

To exclude the interference of ICP subtypes with the outcome of pregnancy, serum TBA levels (Bile acid peak) in pregnant women in each ICP group were quantified and their association with outcome was assessed. This revealed that neonatal weight decreased in step with the increase of TBA level in pregnant women in each of the ICP groups. In ICP1, the incidence of MSAF was significantly higher in women with 40–99 μmol/L TBA than in those with 10–39 μmol/L TBA; the incidence of MSAF and the NICU transfer rate were the highest in pregnant women with ≥ 100 μmol/L TBA. In contrast, in the ICP2 group, the NICU transfer rate gradually rose as the TBA level increased, and the incidence of MSAF and proportion of neonates with an Apgar score ≤ 7 at 1 min was significantly higher in pregnant women with 40–99 μmol/L TBA than with 10–39 μmol/L TBA. In the ICP3 group, the proportions of MSAF cases, neonates transferred to the NICU and neonates with an Apgar score ≤ 7 at 1 min were the highest in pregnant women with ≥ 100 μmol/L TBA. Finally, in the ICP4 group, the proportions of MSAF cases and neonates transferred to the NICU were also the highest in pregnant women with ≥ 100 μmol/L TBA. It can be seen that the pregnancy outcome for patients with different subtypes of ICP was related to the level of TBA. The higher the TBA level (40–99 μmol/L or ≥ 100 μmol/L), the worse the pregnancy outcome (Table S5, Fig. 3).

## Discussion

The maternal serum TBA level has been widely used for clinical diagnosis of ICP and ICP woman was classified by the Society for Maternal–Fetal Medicine (SMFM) (2021) into three risk levels based on the maternal serum TBA level, (6) and was classified into mild type, moderate type and severe type by the Royal College of Obstetricians and Gynecologists (RCOG) Green-top Guidelines (2022). (7) In the present study, we also showed that with the increase in serum TBA level in pregnant women with ICP, the risk of adverse pregnancy outcomes (defined as MSAF, NICU transfer, and Apgar score ≤ 7 at 1 min) significantly rose in step, and the pregnancy outcomes was the worst when TBA was ≥ 100 μmol/L.

UDCA is the first-line drug of choice for the treatment of ICP at present^8^. Manna et al. found that UDCA accounts for approximately 60% of bile acid measurements and can reduce pathological bile acid levels in women receiving treatment^13^. However, the recent PITCHES trial, which compared the effects of UDCA and placebo on ICP outcomes, did not reduce TBA with UDCA, but perhaps related to the fact that PITCHES recruited more women with mild disease than women with severe disease^14^. Therefore, there is an unmet need to comprehensively evaluate the fetal risk and the effect of drug therapy not only on the basis of the serum TBA level in pregnant women with ICP.

In our previous small amount of ICP analysis, (11) ICP pregnant women were classified into four subtypes according to maternal serum biochemical index (TBA, DBIL, ALT) and each subtype has different biochemical characteristics and bile acid profiles. This study is a larger ICP cohort and help us to understand more about clinical characteristics of four subtypes of ICP. The main difference between ICP1 and ICP2 is whether the pregnant women have jaundice. It is very important to further analyze the relationship between pregnant women with jaundice and fetal outcomes. What is the correlation between total bile acid level and fetal outcome in ICP3 subgroup? What factors affect the fetal outcome in ICP4 subgroup with normal bile acid level and abnormal ALT? What is the correlation between different subtypes of ICP? These questions are very important and may affect the fetal outcome, which is also an interesting question to be answered in this paper. As high bile acid levels may have cumulative toxicity to pregnant women and fetuses, it is speculated that early-onset ICP may be more prone to adverse pregnancy outcomes. Is the relationship between early onset ICP and adverse pregnancy outcomes only related to the onset time? Is it also related to other factors, such as the high level of bile acid in pregnant women and/or pregnant women with jaundice?

In this study, we found that the levels of serum biochemical indicators (TBA, DBIL and ALT) in pregnant women initially diagnosed with subtypes of ICP were constantly changing during pregnancy, and consequently, the subtypes of ICP and its severity also changed. UDCA and/or *S*-adenosylmethionine were commonly used in pregnant women with ICP. During the follow-up period, the serum biochemical indicators of pregnant women changed or even became completely normal. Although the initial diagnosis of ICP3 subtype pregnant women only had increased total bile acid level, while DBIL and ALT were normal, the biochemical indicators of pregnant women might also be transformed into ICP1, ICP2, or ICP4 type during the follow-up. Although the initial diagnosis of ICP4 subtype pregnant women only had elevated ALT level, while TBA and DBIL were normal, the biochemical indicators of the pregnant women might also be transformed into ICP1, ICP2, or ICP3 during the follow-up^11^. Therefore, both HP (ICP3) and idiopathic liver enzyme abnormality (ICP4) are also subtyping of ICP, and need to be carefully followed up. Bile acids profile analysis could be helpful to illustrate the characteristics and make diagnosis for ICP3 and ICP4.

The data from Ovadia et al. showed ROC curves for the association between stillbirth and serum biochemical markers (TBA, ALT, AST, BILI) for singleton pregnancies. The predictive value of TBA for stillbirth is better than ALT,AST and BILI^7^. In our data, the cases of stillbirth are very rare because we treat ICP patients very carefully and we have used ICP subtypes analysis clinically for many years. Our results showed that incidence of adverse pregnancy outcomes (defined as meconium-stained amniotic fluid, NICU transfer, and Apgar score ≤ 7 at 1 min, preterm birth) in patients with ICP1 (icteric type) was significantly higher than ICP2, ICP3 and ICP4 subgroups. Thus, adverse pregnancy outcomes for women with ICP are closely related to the subtype of ICP (icteric type or not). Our results showed that maternal jaundice (Direct BILI level beyond normal) is another important marker of adverse pregnancy outcomes of ICP beside maternal high TBA level.

To exclude the interference of ICP subtypes with the pregnancy outcome, serum TBA levels in pregnant women were quantified in each ICP group and the association with pregnancy outcome was assessed. The results revealed that the pregnancy outcomes were correlated with the TBA level in pregnant women with different subtypes of ICP, and the higher the TBA level (40–99 μmol/L or ≥ 100 μmol/L), the worse the pregnancy outcomes. Therefore, the severity of ICP and the risk of adverse pregnancy outcomes should be comprehensively evaluated based on the serum TBA level and ICP subtype (icteric type or not).

In 1995, Kirkinen et al.^15^ recorded ICP occurring in patients at 13 gestational weeks for the first time. At present, the definition of early-onset ICP remains controversial. The 34th gestational week was considered the critical cutoff of gestational week for the diagnosis of early-onset ICP in one retrospective study^16^, while some investigators have proposed that the 32nd week is more appropriate^17^. However, most have taken the 28th gestational week as the critical cutoff between early- and late-onset ICP^12,18^. In this study, we found that preterm birth rates were higher in early-onset ICP than in late-onset ICP in the ICP1 and ICP3 subtypes. Moreover, the pregnancy outcome was poorer in pregnant women with ICP1 (icteric type) in both early-onset ICP and late-onset ICP groups, indicating that the pregnancy outcome of pregnant women with ICP is closely related to the subtype of ICP (icteric type or not). It can also be seen that, whatever early-onset ICP and late-onset ICP groups, the pregnancy outcome for patients with ICP was related to the level of TBA. The higher the TBA level (40–99 μmol/L or ≥ 100 μmol/L), the worse the pregnancy outcome.

Currently, there is no consensus on whether TBA should be measured on fasting or non-fasting. This study was a retrospective analysis, and bile acid levels were measured on fasting. It has been reported that postprandial bile acids are significantly higher than fasting bile acids^19^. Mitchell et al. found poor sensitivity despite good specificity for fasting bile acids. Peak bile acid is an important risk indicator in ICP, so fasting testing may miss correct risk stratification. From the results of this article, no stillbirth was found in our ICP groups (totally 2017 cases of ICP) since Jan 2014 to Aug 2021, according to our management strategy of ICP. Due to the insufficient total number of cases, the number of cases within each subgroup is insufficient, and further research is needed to expand the sample size.

In conclusion, Maternal TBA level and ICP subtype were the key factors of fetal outcomes whatever early- or late-onset ICP. Among the four subtypes of ICP, ICP1 (with jaundice) has the greatest impact on fetal outcome; Adverse outcomes are closely related to the peak serum TBA level (≥ 100 μmol/L) during pregnancy or ICP subtype (icteric type), which requires more attention clinically.

## Methods

### Study design and population

**Figure 4:**
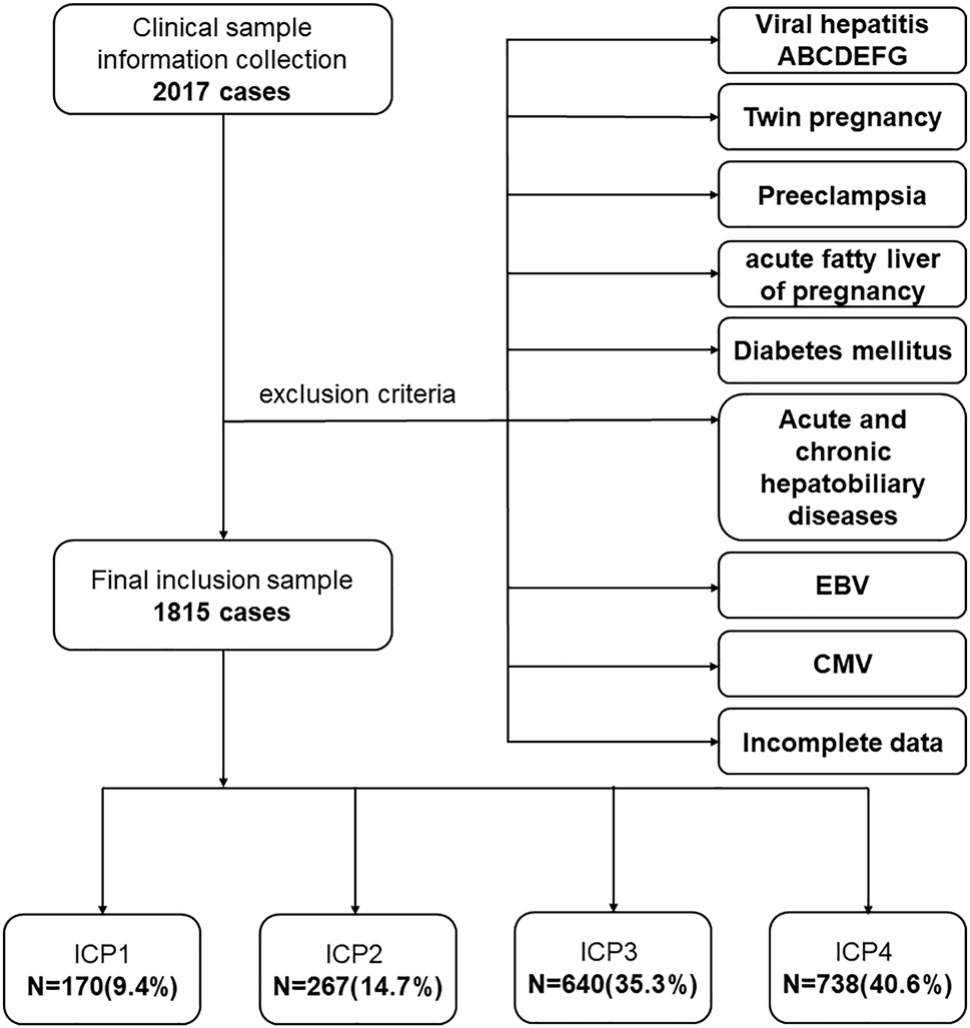
Graphical abstract.

A total of 2017 cases of pregnant women with ICP hospitalized in the First Affiliated Hospital of Chongqing Medical University and Jinshan Branch from January 2014 to August 2021 was reviewed (Fig. 4). After patients with incomplete data, twin pregnancy, viral hepatitis B, preeclampsia, eclampsia, diabetes mellitus, acute and chronic hepatobiliary diseases and other severe complications were excluded, 1815 cases of ICP pregnant women with a singleton pregnancy were finally enrolled (Fig. 1). Informed consent was obtained from all the participants. The pregnancy outcomes of live births were analyzed, including the cesarean section rate, neonatal weight, MSAF, NICU transfer, Apgar score at 1 min, and the gestational age at delivery^20,21^. There were 5 cases of iatrogenic induction of labor, and 4 cases of stillbirth, all of which were vaginal deliveries. Diagnostic criteria: Pregnant women with a serum TBA level ≥ 10 μmol/L, and/or elevated ALT level, and/or DBIL (Above the baseline of normal laboratory reference values). All data were measured on fasting^8^. All ICP pregnant women received UDCA (250 mg tid or qid each day) and/or S-adenosylmethionine (500 mg bid each day) treatment until the serum biochemical indicators were normal or the pregnancy was terminated.

### Procedures and definitions

According to the time of onset, eligible pregnant women with ICP were divided into an early-onset ICP group (time of initial onset < 28 gestational weeks) and a late-onset group (time of initial onset ≥ 28 gestational weeks). According to serum biochemistry, ICP was classified into four subtypes: ICP1 (icteric type, elevated TBA, ALT and DBIL), ICP2 (nonicteric type, elevated TBA and ALT but normal DBIL), ICP3 (hypercholanemia of pregnancy (HP), elevated TBA but normal ALT and DBIL), and ICP4 (idiopathic liver enzyme abnormality in pregnancy, elevated ALT but normal TBA and DBIL)^8^. The proportions of subtypes of ICP including ICP1 (9.4%), ICP2 (14.7%), ICP3 (35.3%) and ICP4 (40.6%) in this cohort are shown in Table 1.

**Table 1:** Biochemical characteristics of ICP subtypes in pregnant women.

Subtype	Cases (n) (%)	TBA	DBIL	ALT	
ICP1	170 (9.4%)	↑	↑	↑	Icteric type
ICP2	267 (14.7%)	↑	N	↑	Nonicteric type
ICP3	640 (35.3%)	↑	N	N	Hypercholanemia of pregnancy
ICP4	738 (40.6%)	N	N	↑	Idiopathic liver enzyme abnormality in pregnancy

Statistical analyses were conducted on the mode of delivery (cesarean section, stillbirth, and iatrogenic induction of labor) and perinatal outcomes (neonatal weight, MSAF, NICU transfer, Apgar score ≤ 7 at 1 min, and time of delivery) in each ICP subgroup. Dynamic changes of maternal serum TBA, DBIL and ALT levels were analyzed in each ICP subgroup during the follow-up period. The pregnancy outcomes of early-onset ICP women were analyzed based on the different levels of serum TBA (0–9.99, 10–39, 40–99, ≥ 100 μmol/L).

### Statistical analysis

SPSS25.0 software was used for data analysis. Measurement data were tested by the rank sum test, *P* < 0.05 was considered statistically significant, and the Bonferroni test was performed for pairwise comparison. The rate and constituent ratio were tested by the chi-square test or Fisher's exact probability test, and the α-partition test was used for pairwise comparison. *P* < 0.017 was considered statistically significant in comparisons among three groups, and *P* < 0.0083 was considered statistically significant in comparisons among four groups.

### Ethics declarations

The present study was conducted following the ethical guidelines established in the 2013 Helsinki Declaration and was approved by the First Affiliated Hospital of Chongqing Medical University (Batch number: 2018-0305).

### Informed consent

Informed consent was obtained from all the participants.

### Supplementary Information

Supplementary Tables.

## Data Availability

The datasets analyzed during the current study are available from the corresponding author on reasonable request.

## Abbreviations

ICP: Intrahepatic cholestasis of pregnancy
MSAF: Meconium-stained amniotic fluid
TBA: Total bile acid
SMFM: Society for Maternal–Fetal Medicine
RCOG: Royal College of Obstetricians and Gynecologists
UDCA: Ursodeoxycholic acid
DBIL: Direct bilirubin
ALT: Alanine aminotransferase
