# Supplementary material for: Associations of clinical subtypes and bile acid levels of intrahepatic cholestasis of pregnancy with pregnancy outcomes
Source: Sci Rep. 2024 May 28;14:12185. doi: 10.1038/s41598-024-63183-9 (PMC11133304; doi:10.1038/s41598-024-63183-9)
Supplement: Supplementary file 1 — Supplementary Tables. [file 41598_2024_63183_MOESM1_ESM.docx]

**Supplemental information titles and legends**

**Table S1 Dynamic changes in ICP subtypes during the follow-up period of pregnant women with ICP**

| **ICP subtypes during the follow-up period** | | | | | | |
| --- | --- | --- | --- | --- | --- | --- |
| **Initial diagnosis of ICP subtype** | **Cases(n)** | **Normal** | **ICP1** | **ICP2** | **ICP3** | **ICP4** |
| **ICP1** | 170 | 15(8.8%) | 126(74.1%) | 25(14.7%) | 18(10.6%) | 28(16.5%) |
| **ICP2** | 267 | 46(17.2%) | 37(13.9%) | 159(59.6%) | 27(10.1%) | 48(18.0%) |
| **ICP3** | 640 | 173(27.0%) | 13(2.0%) | 21(3.3%) | 497(77.7%) | 18(2.8%) |
| **ICP4** | 738 | 170(23.0%) | 97(13.1%) | 259(35.1%.) | 53(7.2%) | 409(55.4%) |

**Table S2 Pregnancy outcomes for women with subtypes of ICP**

|  |  | **ICP1(n=170)** | **ICP2(n=267)** | **ICP3(n=640)** | **ICP4(n=738)** | **P-value** |
| --- | --- | --- | --- | --- | --- | --- |
|  | **Age(year) M(P25,p75)** | 29(26,32) | 27(25,31) | 28(25,32) | 28(26,31) | 0.064 |
| **Mode of delivery (n,%)** | **Cesarean section** | 155(92.3%)^a^ | 226(85.6%)^b^ | 472(74.0%)^c^ | 590(80.0%) | <0.001 |
|  | **Stillbirth** | 1(0.6%) | 1(0.4%) | 2(0.3%) | 0 | 0.144 |
|  | **Iatrogenic induction of labor** | 1(0.6%) | 2(0.7%) | 0 | 2(0.3%) | 0.097 |
| **Pregnancy outcomes(n,%)** | **Neonatal weight (g) M(P25,p75)** | 3065(2730,3298)^d^ | 3080(2784,3398)^e^ | 3160(2868,3433) | 3200(2880,3480) | <0.001 |
|  | **Meconium-stained**  **amniotic fluid** | 56(33.3%)^a^ | 59(22.3%) | 98(15.4%) | 130(17.7%) | <0.001 |
|  | **NICU** | 35(21.0%)^f^ | 28(10.6%) | 42(6.6%) | 52(7.1%) | <0.001 |
|  | **1 min Apgar scores ≤ 7** | 5(3.0%)^a^ | 3(1.1%) | 3(0.5%) | 3(0.4%) | 0.004 |
|  | **1 min Apgar scores** **< 4** | 0 | 0 | 1(0.2%) | 1(0.1%) | 1.000 |
|  | **5 min Apgar scores < 7** | 0 | 0 | 1(0.2%) | 1(0.1%) | 1.000 |
|  | **Preterm birth** | 68(40.5%)^f^ | 62(23.5%)^a^ | 85(13.3%) | 118(46.0%) | <0.001 |
|  | **Spontaneous preterm birth** | 5(3.0%) | 2(0.8%) | 9(1.4%) | 7(1.0%) | 0.162 |
|  | **Iatrogenic preterm birth** | 63(37.5%)^f^ | 60(22.7%)^a^ | 76(11.9%) | 111(15.1%) | <0.001 |
|  | **Time of delivery (d) M(P25，p75)** | 260(253,267)^g^ | 267(259,274)^d^ | 270(264,276) | 269(261,276) | <0.001 |

^a^ Compared with ICP3 and ICP4 group，*P＜*0.0083

^b^ Compared with ICP3 group，*P＜*0.0083

^c^ Compared with ICP4 group，*P＜*0.0083

^d^ Compared with ICP3 and ICP4 group，adj.*P＜*0.05

^e^ Compared with ICP4 group，adj.*P＜*0.05

^f^ Compared with ICP2, ICP3 and ICP4 group，*P＜*0.0083

^g^ Compared with ICP2, ICP3 and ICP4 group，adj.*P＜*0.05

**Table S3** **Pregnancy outcomes for women with different subtypes of early- or late-onset ICP**

|  | **Subjects** | **ICP1** | **ICP2** | **ICP3** | **ICP4** | **P-value** |
| --- | --- | --- | --- | --- | --- | --- |
| **Time of initial onset <28 gestational weeks** | **Cases(n)** | 23(5.7%) | 37(9.3%) | 169(42.5%) | 169(42.5%) |  |
|  | **Cesarean section (n,%)** | 20(90.9%) | 31(88.6%) | 132(78.1%) | 136(81.0%) | 0.305 |
|  | **Stillbirth (n,%)** | 0 | 1(2.7%) | 0 | 0 | 0.151 |
|  | **Iatrogenic induction of labor (n,%)** | 1(4.3%) | 1(2.7%) | 0 | 1(0.6%) | 0.061 |
|  | **Neonatal weight (g)M(P25,p75)** | 2830(2500,3080)^a^ | 3010(2635,3480) | 3100(2840,3400) | 3160(2880,3510) | <0.001 |
|  | **Meconium-stained**  **amniotic fluid (n,%)** | 11(50.0%)^b^ | 10(28.6%) | 24(14.3%) | 32(19.2%) | 0.001 |
|  | **NICU(n,%)** | 7(31.8%)^b^ | 8(22.9%) | 14(8.3%) | 15(8.9%) | 0.001 |
|  | **1 min Apgar scores ≤ 7 (n,%)** | 0 | 0 | 2(1.2%) | 1(0.6%) | 1.000 |
|  | **Preterm birth** | 14(63.6%)^b^ | 12(34.3%) | 37(21.9%) | 35(20.8%) | <0.001 |
|  | **Spontaneous preterm birth** | 2(9.1%) | 0 | 3(1.8%) | 3(1.8%) | 0.096 |
|  | **Iatrogenic preterm birth** | 12(54.5%)^b^ | 12(34.3%) | 34(20.1%) | 32(19.0%) | 0.001 |
|  | **Time of delivery (d)M(P25,p75)** | 256(249,260)^c^ | 264(254,276) | 267(259,275) | 268(260,276) | <0.001 |
| **Time of initial onset ≥28 gestational weeks** | **Cases(n)** | 147(10.4%) | 230(16.2%) | 471(33.2%) | 569(40.2%) |  |
|  | **Cesarean section (n,%)** | 135(92.5%)^b^ | 195(85.2%)^d^ | 340(72.5%)^e^ | 454(79.9%) | <0.001 |
|  | **Stillbirth (n,%)** | 1(0.7%) | 0 | 2(0.4%) | 0 | 0.168 |
|  | **Iatrogenic induction of labor (n,%)** | 0 | 1(0.4%) | 0 | 1(0.2%) | 0.572 |
|  | **Neonatal weight (g)M(P25,p75)** | 3100(2750,3335)^a^ | 3100(2800,3390) | 3200(2870,3450) | 3200(2880,3480) | 0.003 |
|  | **Meconium-stained**  **amniotic fluid (n,%)** | 45(30.8%)^b^ | 49(21.4%) | 74(15.8%) | 98(17.3%) | <0.001 |
|  | **NICU(n,%)** | 28(19.3%)^f^ | 20(8.8%) | 28(6.0%) | 37(6.5%) | <0.001 |
|  | **1 min Apgar scores ≤ 7 (n,%)** | 5(3.4%)^b^ | 3(1.3%) | 1(0.2%) | 2(0.4%) | 0.001 |
|  | **Preterm birth** | 54(37.0%)^f^ | 50(21.8%)^d^ | 48(10.2%) | 83(14.6%) | <0.001 |
|  | **Spontaneous preterm birth** | 3(2.1%) | 2(0.9%) | 6(1.3%) | 4(0.7%) | 0.503 |
|  | **Iatrogenic preterm birth** | 51(34.9%)^f^ | 48(21.0%)^d^ | 42(9.0%) | 79(13.9%) | <0.001 |
|  | **Time of delivery (d)M(P25,p75)** | 260(255,268)^c^ | 267(259,273)^a^ | 271(265,277) | 270(262,276) | <0.001 |

^a^ Compared with ICP3 and ICP4 group, adj.*P＜*0.05

^b^ Compared with ICP3 and ICP4 group, *P＜*0.0083

^c^ Compared with ICP2, ICP3 and ICP4 group, adj.*P＜*0.05

^d^ Compared with ICP3 group, *P＜*0.0083

^e^ Compared with ICP4 group, *P＜*0.0083

^f^ Compared with ICP2, ICP3 and ICP4 group，*P＜*0.0083

**Table S4** **Association between gestational week and pregnancy outcome for pregnant women with early- or late-onset ICP**

|  | **Subjects** | **Early-onset ICP** | **Late-onset ICP** | **P-value** |
| --- | --- | --- | --- | --- |
| **ICP1(n=170)** | **Cesarean section (n,%)** | 20(90.9%) | 135(92.5%) | 1.000 |
|  | **Stillbirth (n,%)** | 0 | 1(0.7%) | 1.000 |
|  | **Iatrogenic induction of labor (n,%)** | 1(4.3%) | 0 | 0.135 |
|  | **Neonatal weight (g)M(P25,p75)** | 2830(2500,3080) | 3100(2750,3335) | 0.003 |
|  | **Meconium-stained**  **amniotic fluid (n,%)** | 11(50.0%) | 45(30.8%) | 0.075 |
|  | **NICU(n,%)** | 7(31.8%) | 28(19.3%) | 0.288 |
|  | **1 min Apgar scores ≤ 7 (n,%)** | 0 | 5(3.4%) | 1.000 |
|  | **Preterm birth** | 14(63.3%) | 54(37.0%) | 0.018 |
|  | **Spontaneous preterm birth** | 2(9.1%) | 3(2.1%) | 0.255 |
|  | **Iatrogenic preterm birth** | 12(54.5%) | 51(34.9%) | 0.076 |
|  | **Time of delivery (d)M(P25,p75)** | 256(249,260) | 260(255,268) | 0.003 |
| **ICP2(n=267)** | **Cesarean section (n,%)** | 31(88.6%) | 195(85.2%) | 0.592 |
|  | **Stillbirth (n,%)** | 1(2.7%) | 0 | 0.139 |
|  | **Iatrogenic induction of labor (n,%)** | 1(2.7%) | 1(0.4%) | 0.258 |
|  | **Neonatal weight (g)M(P25,p75)** | 3010(2635,3480) | 3100(2800,3390) | 0.439 |
|  | **Meconium-stained**  **amniotic fluid (n,%)** | 10(28.6%) | 49(21.4%) | 0.343 |
|  | **NICU(n,%)** | 8(22.9%) | 20(8.8%) | 0.026 |
|  | **1 min Apgar scores ≤ 7 (n,%)** | 0 | 3(1.3%) | 1.000 |
|  | **Preterm birth** | 12(34.3%) | 50(21.8%) | 0.106 |
|  | **Spontaneous preterm birth** | 0 | 2(0.9%) | 1.000 |
|  | **Iatrogenic preterm birth** | 12(34.3%) | 48(21.0%) | 0.080 |
|  | **Time of delivery (d)M(P25,p75)** | 264(254,276) | 267(259,273) | 0.334 |
| **ICP3(n=640)** | **Cesarean section (n,%)** | 132(78.1%) | 340(72.5%) | 0.154 |
|  | **Stillbirth (n,%)** | 0 | 2(0.4%) | 1.000 |
|  | **Iatrogenic induction of labor (n,%)** | 0 | 0 |  |
|  | **Neonatal weight (g)M(P25,p75)** | 3100(2840,3400) | 3200(2870,3450) | 0.030 |
|  | **Meconium-stained**  **amniotic fluid (n,%)** | 24(14.3%) | 74(15.8%) | 0.645 |
|  | **NICU(n,%)** | 14(8.3%) | 28(6.0%) | 0.298 |
|  | **1 min Apgar scores ≤ 7 (n,%)** | 2(1.2%) | 1(0.2%) | 0.173 |
|  | **Preterm birth** | 37(21.9%) | 48(10.2%) | <0.001 |
|  | **Spontaneous preterm birth** | 3(1.8%) | 6(1.3%) | 0.930 |
|  | **Iatrogenic preterm birth** | 34(20.1%) | 42(9.0%) | <0.001 |
|  | **Time of delivery (d)M(P25,p75)** | 267(259,275) | 271(265,277) | <0.001 |
| **ICP4(n=738)** | **Cesarean section (n,%)** | 136(81.0%) | 454(79.9%) | 0.770 |
|  | **Stillbirth (n,%)** | 0 | 0 |  |
|  | **Iatrogenic induction of labor (n,%)** | 1(0.6%) | 1(0.2%) | 0.406 |
|  | **Neonatal weight (g)M(P25,p75)** | 3160(2880,3510) | 3200(2880,3480) | 0.899 |
|  | **Meconium-stained**  **amniotic fluid (n,%)** | 32(19.2%) | 98(17.3%) | 0.576 |
|  | **NICU(n,%)** | 15(8.9%) | 37(6.5%) | 0.283 |
|  | **1 min Apgar scores ≤ 7 (n,%)** | 1(0.6%) | 2(0.4%) | 0.541 |
|  | **Preterm birth** | 35(20.8%) | 83(14.6%) | 0.054 |
|  | **Spontaneous preterm birth** | 3(1.8%) | 4(0.7%) | 0.414 |
|  | **Iatrogenic preterm birth** | 32(19.0%) | 79(13.9%) | 0.102 |
|  | **Time of delivery (d)M(P25,p75)** | 268(260,276) | 270(262,276) | 0.132 |

**Table S5 Association between serum TBA level and pregnancy outcome**

|  | **Subjects** | **0-9 μmol/L (TBA)** | **10-39 μmol/L (TBA)** | **40-99 μmol/L (TBA)** | **≥100 μmol/L (TBA)** | **P-value** |
| --- | --- | --- | --- | --- | --- | --- |
| **ICP1** | **Cases(n)** | 0 | 86 | 58 | 26 |  |
|  | **Cesarean section (n,%)** | 0 | 78(90.7%) | 56(96.6%) | 21(87.5%) | 0.279 |
|  | **Stillbirth (n,%)** | 0 | 0 | 0 | 1(3.8%) | 0.153 |
|  | **Iatrogenic induction of labor (n,%)** | 0 | 0 | 0 | 1(3.8%) | 0.153 |
|  | **Neonatal weight (g)M(P25,p75)** | 0 | 3208(2833,3460) | 2940(2735,3163)^a^ | 2655(2400,2868)^b^ | <0.001 |
|  | **Meconium-stained**  **amniotic fluid (n,%)** | 0 | 21(24.4%) | 24(41.4%) | 11(45.8%) | 0.040 |
|  | **NICU(n,%)** | 0 | 14(16.3%) | 11(19.3%) | 10(41.7%)^c^ | 0.024 |
|  | **1 min Apgar scores ≤ 7 (n,%)** | 0 | 2(2.3%) | 1(1.7%) | 2(8.3%) | 0.280 |
|  | **Time of delivery (d)M(P25,p75)** | 0 | 264(258,271) | 259(253,263)^a^ | 252(245,259)^a^ | <0.001 |
| **ICP2** | **Cases(n)** | 0 | 197 | 55 | 15 |  |
|  | **Cesarean section (n,%)** | 0 | 160(81.6%) | 51(96.2%)^c^ | 15(100.0%) | 0.007 |
|  | **Stillbirth (n,%)** | 0 | 0 | 1(1.8%) | 0 | 0.262 |
|  | **Iatrogenic induction of labor (n,%)** | 0 | 1(0.5%) | 1(1.8%) | 0 | 0.456 |
|  | **Neonatal weight (g)M(P25,p75)** | 0 | 3135(2838,3440) | 3010(2805,3345) | 2420(2140,2670)^b^ | <0.001 |
|  | **Meconium-stained**  **amniotic fluid (n,%)** | 0 | 34(17.3%) | 22(41.5%)^c^ | 3(20.0%) | 0.001 |
|  | **NICU(n,%)** | 0 | 12(6.2%) | 9(17.0%)^c^ | 7(46.7%)^c^ | <0.001 |
|  | **1 min Apgar scores ≤ 7 (n,%)** | 0 | 0 | 3(5.7%)^c^ | 0 | 0.023 |
|  | **Time of delivery (d)M(P25,p75)** | 0 | 268(261,274) | 263(254,273)^a^ | 252(245,258)^b^ | <0.001 |
| **ICP3** | **Cases(n)** | 0 | 492 | 129 | 19 |  |
|  | **Cesarean section (n,%)** | 0 | 332(67.8%) | 123(95.3%)^c^ | 17(89.5%) | <0.001 |
|  | **Stillbirth (n,%)** | 0 | 2(0.4%) | 0 | 0 | 1.000 |
|  | **Iatrogenic induction of labor (n,%)** | 0 | 0 | 0 | 0 |  |
|  | **Neonatal weight (g)M(P25,p75)** | 0 | 3230(2960,3470) | 2935(2678,3208)^a^ | 2665(2491,3244)^a^ | <0.001 |
|  | **Meconium-stained**  **amniotic fluid (n,%)** | 0 | 73(14.9%) | 18(14.1%) | 7(36.8%)^d^ | 0.030 |
|  | **NICU(n,%)** | 0 | 17(3.5%) | 18(14.0%)^c^ | 7(36.8%)^d^ | <0.001 |
|  | **1 min Apgar scores ≤ 7 (n,%)** | 0 | 2(0.4%) | 0 | 1(5.3%) | 0.095 |
|  | **Time of delivery (d)M(P25,p75)** | 0 | 272(267,277) | 263(255,269)^a^ | 253(250,261)^a^ | <0.001 |
| **ICP4** | **Cases(n)** | 390 | 291 | 49 | 8 |  |
|  | **Cesarean section (n,%)** | 298(76.8%) | 238(81.8%) | 47(95.9%)^e^ | 7(87.5%) | 0.011 |
|  | **Stillbirth (n,%)** | 0 | 0 | 0 | 0 |  |
|  | **Iatrogenic induction of labor (n,%)** | 2(0.5%) | 0 | 0 | 0 | 0.583 |
|  | **Neonatal weight (g)M(P25,p75)** | 3260(2940,3540) | 3170(2850,3440) | 3030(2590,3275)^f^ | 2918(2588,3200) | <0.001 |
|  | **Meconium-stained**  **amniotic fluid (n,%)** | 56(14.5%) | 56(19.3%) | 14(28.6%) | 4(50.0%)^e^ | 0.005 |
|  | **NICU(n,%)** | 15(3.9%) | 22(7.6%) | 12(24.5%)^g^ | 3(37.5%)^e^ | <0.001 |
|  | **1 min Apgar scores ≤ 7 (n,%)** | 0 | 1(0.3%) | 2(4.1%) | 0 | 0.015 |
|  | **Time of delivery (d)M(P25,p75)** | 272(266,278) | 267(260,274)^h^ | 260(251,269)^f^ | 255(248,256)^f^ | <0.001 |

^a^ Compared with 10-39.99 μmol/L (TBA)，adj.*P＜*0.05

^b^ Compared with 10-39.99 and 40-99.99 μmol/L (TBA)，adj.*P＜*0.05

^c^ Compared with 10-39.99 μmol/L (TBA)，*P＜*0.017

^d^ Compared with 10-39.99 and 40-99.99 μmol/L (TBA)，*P＜*0.017

^e^ Compared with 0-9.99 μmol/L (TBA)，*P＜*0.0083

^f^ Compared with 0-9.99 and 10-39.99 μmol/L (TBA)，adj.*P＜*0.05

^g^ Compared with 0-9.99 and 10-39.99 μmol/L (TBA)，*P＜*0.0083

^h^ Compared with 0-9.99 μmol/L (TBA)，adj.*P＜*0.05
